# Changes in the Molecular Phenotype of Nucleus Pulposus Cells with Intervertebral Disc Aging

**DOI:** 10.1371/journal.pone.0052020

**Published:** 2012-12-19

**Authors:** Xinyan Tang, Liufang Jing, Jun Chen

**Affiliations:** 1 Department of Biomedical Engineering, Duke University, Durham, North Carolina, United States of America; 2 Department of Orthopedic Surgery, Duke University Medical Center, Durham, North Carolina, United States of America; National University of Ireland, Galway, Ireland

## Abstract

Intervertebral disc (IVD) disorder and age-related degeneration are believed to contribute to low back pain. Cell-based therapies represent a promising strategy to treat disc degeneration; however, the cellular and molecular characteristics of disc cells during IVD maturation and aging still remain poorly defined. This study investigated novel molecular markers and their age-related changes in the rat IVD. Affymetrix cDNA microarray analysis was conducted to identify a new set of genes characterizing immature nucleus pulposus (NP) cells. Among these markers, select neuronal-related proteins (Basp1, Ncdn and Nrp-1), transcriptional factor (Brachyury T), and cell surface receptors (CD24, CD90, CD155 and CD221) were confirmed by real-time PCR and immunohistochemical (IHC) staining for differential expression between IVD tissue regions and among various ages (1, 12 and 21 months). NP cells generally possessed higher levels of mRNA or protein expression for all aforementioned markers, with the exception of CD90 in anulus fibrosus (AF) cells. In addition, CD protein (CD24 and CD90) and Brachyury (T) expression in immature disc cells were also confirmed via flow cytometry. Similar to IHC staining, results revealed a higher percentage of immature NP cells expressing CD24 and Brachyury, while higher percentage of immature AF cells was stained positively for CD90. Altogether, this study identifies that tissue-specific gene expression and age-related differential expression of the above markers do exist in immature and aged disc cells. These age-related phenotype changes provide a new insight for a molecular profile that may be used to characterize NP cells for developing cell-based regenerative therapy for IVD regeneration.

## Introduction

The human intervertebral disc (IVD), a heterogeneous soft tissue that lies in the space between adjacent vertebral bodies, provides flexibility and load support in the spine [1]. Significant cell-mediated tissue remodeling occurs in the IVD as a consequence of aging, marked by an increasingly fibrotic nucleus pulposus (NP), disoriented lamellae in the annulus fibrosus (AF), and calcified vertebral endplates [2]. These age-related changes may lead to IVD degenerative disorders, such as internal disc disruption, AF tears, and “herniated” or “extruded” NP [3]. These anatomic features can be associated with symptoms of low back pain, neurological deficits, and disability that affect 30% of the US population annually [4], [5]. Current treatments for disc disorders merely offer temporary symptom relief, and cannot restore original structure and function. Although several therapeutic advances have been demonstrated in animal models [6], [7], a more thorough understanding of molecular phenotype changes in the NP cell population during aging will surely catalyze the development of cell-based therapies for IVD regeneration.

Multiple cell populations that are morphologically and biosynthetically distinct exist within the IVD. The AF is populated by fibrochondrocyte-like cells of mesenchymal origin [8], while the NP consists of a mixture of small chondrocyte-like mesenchymal cells and larger notochordal-derived cells [9], [10], [11]. In neonatal and immature tissues, NP cells are large and highly vacuolated, appearing in clusters with tight cell-to-cell connections and a dense cytoskeletal network [12], [13], [14]. As the IVD matures, there is a morphological shift in the population of these larger and highly vacuolated cells (often called “notochordal cells” to reflect their origin in the notochord) towards smaller fibrochondrocyte-like cells [15]. However, molecular phenotype changes with age progression still remain unclear. The well-hydrated gelatinous matrix formed by NP cells is conducive for preserving disc height, biomechanical function and the homeostasis of the IVD microenvironment [16], [17], [18], [19], [20]. Unfortunately, aging proves detrimental for cell survival and thus leads to decreased cell density and matrix synthesis [21], [22], [23]. Notochordal cells of the immature NP may play important stimulatory roles that promote matrix biosynthesis in other disc cell types [24], [25]. Hence, the process of notochordal cell disappearance during aging has been suggested to initiate a metabolic imbalance in the IVD that may contribute to IVD degeneration [26]. In human and chondrodystrophoid species of dog, loss of these notochordal cells coincides with the onset of disc degeneration [24]. Although the precise mechanism and functionality of disappearance of notochordal cells in NP remains poorly understood, notochordal cells have generated substantial interest due to their posited role in generating and maintaining proteoglycan-rich, functional NP tissue. Further understandings of the molecular cell phenotype (i.e. molecular markers of NP cell phenotype) may be useful in developing cellular therapies for NP regeneration, as well as for identifying specific soluble factors produce by these cells which stimulate the disc’s existing cells for matrix regeneration.

Our previous study demonstrated that specific laminin isoforms (LM511 and LM322), laminin receptors (CD239 and integrin subunits α3, β1, α6, and β4) are highly expressed in NP as compared to AF [27], [28]. Additionally, it was noted that immature NP cells exhibit unique cell-laminin interaction for maintaining notochordal cell morphology [29]. Many other studies have also focused on identification of unique markers for NP or AF cells to better characterize cell phenotype. It has been reported that mRNA or protein for HIF-1α, GLUT-1, MMP-2, CD24, CD44, CD56, CD151, glypican3, cytokeratin 8, 18 and 19, CDH2, SNAP25, BSAP1 and FOXF1 were highly expressed in NP as compared to AF [27], [30], [31], [32], [33], [34], [35]. Many of these studies evaluated the differential expression between AF and NP regions, yet it remains unclear whether these genes can be used as markers to define disc cell phenotypes and to distinguish NP cells from AF cells during aging. The objective of this study was thereby to elucidate changes in a new set of molecular markers during aging in the rat IVD. cDNA microarray was employed to screen markers based on functions, including three neuronal-related proteins (brain abundant membrane attached signal protein 1, Basp1; Neurochondrin, Ncdn; Neuropilin, Nrp-1), a transcriptional factor (Brachyury T) and cell surface proteins (CD24, CD221, CD155 and CD90). Real time RT-PCR and immunohistochemistry were used to confirm marker expression profiles at both gene and protein levels respectively. In addition, flow cytometry was performed to analyze the protein expression of two cell surface receptors, CD24, and CD90, and a transcriptional factor, Brachyury T, in primary cells isolated from immature NP and AF tissues. Examining marker expression in immature NP and their age-related changes should enable better characterization of NP cells, which may be applied toward evaluating IVD degeneration and developing effective cell-based therapies.

## Results

### Cell Morphological Changes in the Aging NP

**Figure 1:**
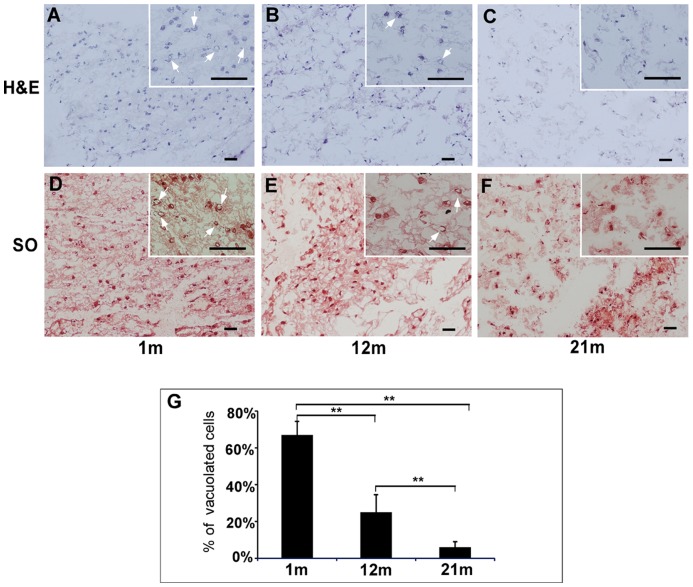
Histological characterization of NP tissue from IVD of rats at different ages. Frozen tissue sections from (A, D) 1 m, (B, E) 12 m and (C, F) 21 m-old rats were stained with (A, B, C) Hematoxylin and Eosin (H&E) for cell structure and (D, E, F) Safranin O (SO) for proteoglycan content. Representative images are shown and the inserts of small images shown higher magnifications of interested regions. Bar: 50 µm. m: month. Arrows in subfigures indicate vacuolated morphology. (G) The percentage of cells with vacuolated morphology indicates an age-dependent change in NP tissue. **p<0.01 as compared to 1 m NP (one-factor ANOVA, n = 8). (TIF)

Both H&E and Safranin O staining clearly illustrated differences in cellular structure and extracellular matrix for NP tissue among different ages (1 m, 12 m and 21 m, Fig. 1). In general, the NP tissues exhibited an age-dependent decrease in total cell number. Moreover, a significant higher percentage (∼67%) of cells with vacuole-like structure was observed in immature NP (1 m, Fig. 1 A, D, G,) as compared to that in mature and aged NP. About 25% of cells with vacuole-like structures were still observed in the mature NP (12 m, Fig. 1 B, E, G), but only 6% was kept in the aged NP (21 m, Fig. 1 C, F, G). This finding of an age-dependent decrease in vacuole-like cells remains consistent with previous reports of sparse or no notochordal cells found in 1 to 2-year old rats [9], [36]. Additionally, Safranin O staining revealed that 1 m NP tissue displayed a denser matrix structure (Fig. 1 D) compared with 12 m and 21 m NP, which were shown to be more scattered and clustered (Fig. 1 E, F).

### Gene Expression Profile of the NP and AF

A comparative gene expression analysis using the Affymetrix GeneChip (Rat Genome Array) was performed to identify novel genes that may uniquely distinguish the NP from the AF. The expressed gene profile of all genes, transcript variants and ESTs (expressed sequence tags) (total of 31,099 substantiated rat genes) in the rat IVD was evaluated, and potential NP markers were selected based on those molecules exhibiting higher mRNA expression levels in immature NP as compared to AF. Similarly, changes in mRNA levels of these markers during aging were also assessed between immature (1 m) and mature (12 m) NP. Results indicated that ∼2% of genes (∼644 transcripts) exhibited differences with aging, and ∼12% of genes (∼ 3668 transcripts) exhibited differences with tissue type (NP vs. AF). Table 1 contains a partial list of target genes differentially expressed between NP and AF tissue (1 m) and between immature and mature NP (1 m and12 m). These target genes related to NP cell functions were grouped into three categories: cell surface receptors, transcriptional factors and neuronal-related proteins.

**Table 1:** Average fold-differences in relative mRNA levels of selected genes between both tissue regions (AF & NP of 1 m rat) and ages (1 m & 12 m of NP) analyzed by cDNA microarray (n = 4, p<0.01, fold-differences greater than or equal to 2 folds between groups by one-factor ANOVA).

Gene name	Gene symbol	Ref. sequence	Fold-difference	
			**in region**	**in age**
			1 m NP vs.	1m NP vs.
			1 m AF^a^	12 m NP^b^
**Cell surface receptor**				
Lutheran (Lu)	LU, CD239,	NM_031752	10.2	–
	BCAM			
Activated leukocyte cell adhesion molecule	CD166, ALCAM	NM_031753	6.6	–
Tumor necrosis factor receptor superfamily,	TNFRSF12A	NM_181086	5.0	–
member 12a				
**Poliovirus receptor tumor associate antigen 1**	**PVR, CD155**	NM_017076	4.5	2.0
Cadaherin 2	CDH2, CD325	NM_031333	4.3	2.0
intercellular adhesion molecule 1	ICAM, CD54	NM_012967	3.8	–
Tetraspanin, CD151	CD151, PETA-3	NM_022523	3.6	–
**Insulin-like growth factor-1 receptor**	**IGF-1R, CD221**	NM_052807	3.4	–
Aiscoidin domain receptor tyrosine kinase 1	DDR1, CD167	NM_001166022	3.2	–
**CD24**	**CD24**	NM_012752	2.8	–
Syndecan4	SDC4	NM_012649	2.6	–
Hyaluronan mediated motility receptor	CD168/RHAMM	NM_012964	2.6	2.6
Galectin-1	LGAS1, GAL-1	NM_019904	3.1	2.5
Lysosomal-associated membrane protein 1	CD107a/Lamp1	NM_012857	2.1	–
Syndecan2	SDC2, CD362	NM_013082	−3.8	–
**Thy-1 cell surface antigen**	**THY1, CD90**	NM_012673	−4.5	4.6
Syndecan1	SDC1, CD138	NM_013026	−7.1	2.7
**Transcriptional factors**				
Nuclear receptor subfamily 3, group C, member 2	NR3C2	NM_013131	4.1	–
**BrachyuryT2** ^c^	**T2**	NM_001161835	2.7	–
Sterol regulatory element binding transcription	SREBF2	NM_001033694	2.4	–
factor 2				
Kruppel-like factor 6	KLF6	NM_031642	2.4	–
Sterol regulatory element binding transcription	SREBF1	XM_001075680	2.3	–
factor 1				
V-rel reticuloendotheliosis viral oncogene	RELa	NM_199267	2.1	–
homolog A (avian)				
Hypoxia-inducible factor 1α	HIF1A	NM_024359	−2.0	–
Inhibitor of DNA binding 3, dominant negative	ID3	AF000942	−2.1	5.2
helix-loop-helix protein				
Forkhead box A2	FOXA2, HNF3B	NM_012743	−4.1	–
Runt-related transcription factor 1	RUNX1	NM_017325	−5.4	–
Nuclear factor I/B	NFIb	NM_031566	−5.5	–
Inhibitor of DNA binding 2, dominant negative	ID2	NM-013060	−7.2	3.9
helix-loop-helix protein				
CCAAT/enhancer binding protein (C/EBP), beta	CEBPb	NM_024125	−7.7	–
**Neuronal-related proteins**				
**Neurochondrin**	**NCDN**	NM_053543	9.4	2.0
Leucine rich repeat protein 3	LRRN3	NM_030856	8.0	3.1
Dipeptidylpeptidase 6	DPP6	NM_022850	6.5	2.0
A5D3 protein	AARD	NM_145093	5.8	12.4
**Brain abundant membrane signal protein 1**	**BASP1**	NM_22300	4.8	–
Necdin	NDN	NM_001008558	4.0	2.6
**Neuropilin-1**	**NRP-1**	NM_012613	4.0	3.6

a. Fold-differences were nomalized to 1 m AF; b. Fold-differences were nomalized to 12 m NP; c. Brachyury T gene was not included in the rat genome 230 2.0 Array, therefore T2 was listed here. Bolded gene names/symbles indicate the targets selected for PCR and protein expression anylsis. –: fold not changed; m: month.

#### Gene expression of cell surface receptors in IVD tissues

As shown in Table 1, results confirmed higher mRNA expression levels in immature rat NP as compared to AF for several cell surface receptors CD239 (Lu), CD151 (PETA-3), CD24, CD54 (ICAM), CD325 (CDH2) and galectin-1(GAL-1) identified in previous studies of rat, porcine, bovine or human IVDs [27], [30], [33], [37], [38]. mRNA levels for other receptors highly expressed in NP tissues were ALCAM (CD166), TNFRSF12A, PVR (CD155), IGF-1R (CD221), DDR1(CD167), SDC4, RHAMM (CD168) and LAMP1(CD107a). Additional findings in Table 1 illustrated lower levels for CD90 (THY1), SDC2 (CD362), SDC1 (CD138) in the immature NP as compared to AF. Immature NP also expressed higher mRNA levels of six receptors or proteins (CD155, CD325, CD168, CD90, CD138 and GAL-1) as compared to mature NP (see Table 1).

#### Gene expression of transcriptional factors in IVD tissues

Results in Table 1 shown thirteen transcriptional factors that were differentially expressed between tissue regions or ages. mRNA levels for six transcriptional factors (NR3C2, T2, SREBF2, KLF6, SREBF1 and RELa) were higher in immature NP as compared to AF regions, while mRNA levels for another seven transcriptional factors (HIF1A, ID3, FOXA2, RUNX1, NFIb, ID2 and CEBPb) were higher in immature AF as compared to NP regions. In addition, immature NP also expressed higher mRNA levels of transcriptional factors ID3 and ID2 as compared to mature NP.

#### Gene expression of neuronal-related proteins in IVD tissues

Seven genes for neuronal-related proteins (NCDN, LRRN3, DPP, AARD, BASP1, NDN and NRP-1) were found to be highly expressed in immature NP tissues as compared to AF regions (Table 1). With the exception of BASP1, six other genes were also highly expressed in immature NP as compared to mature NP.

### Selected NP Markers Expression during Aging Confirmed by Real Time RT-PCR

**Figure 2:**
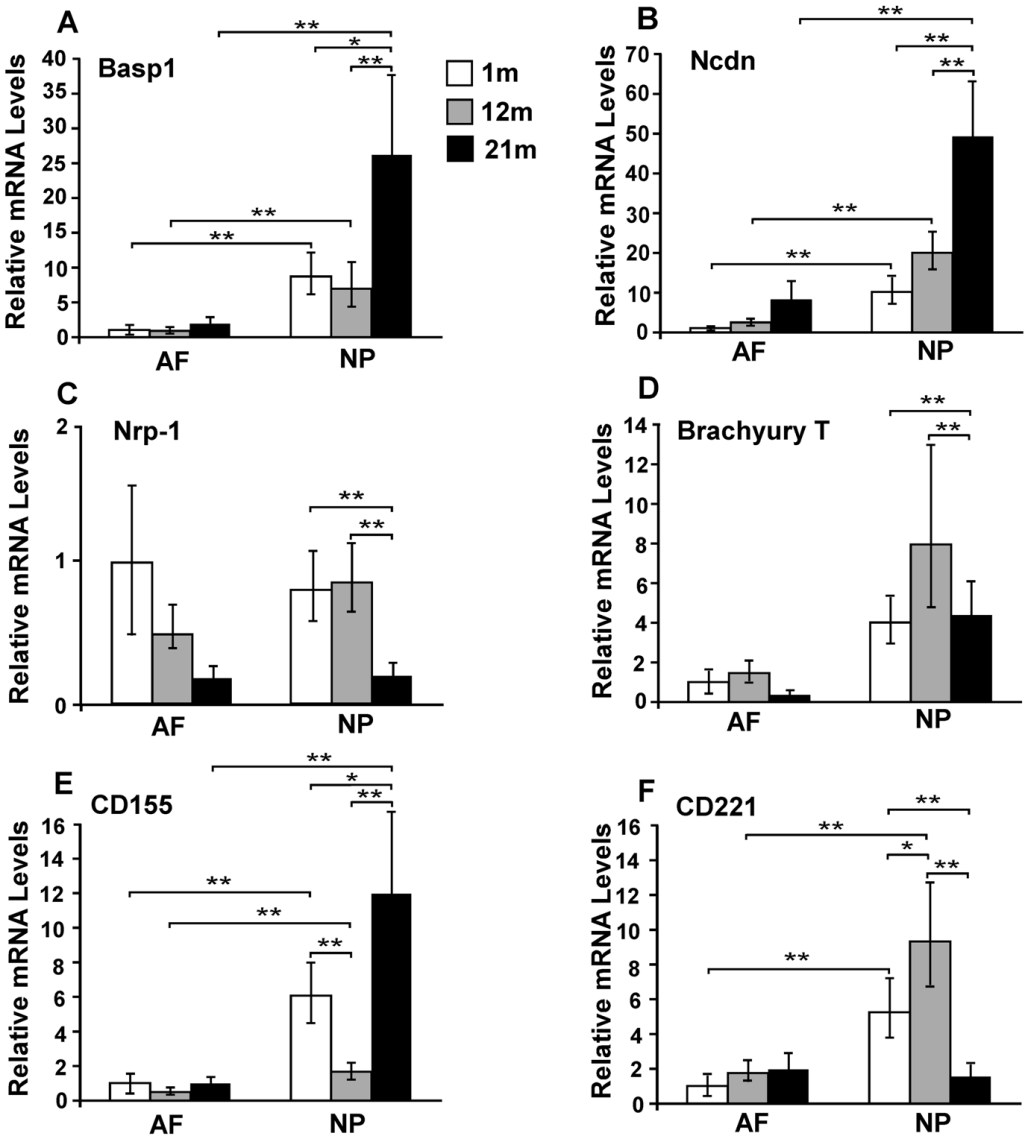
Realtime RT-PCR for relative mRNA level (2^−ΔΔCt^) of NP markers. (A) Basp1; (B) Ncdn; (C) Nrp-1; (D) Brachyury T; (E) CD155; (F) CD221 in rat IVD tissues at different ages (1 m, 12 m and 21 m). All values of fold-difference were normalized to AF tissue (1 m) for comparison between different tissue regions and ages. m: month. * p<0.05, ** p<0.01, two-factor ANOVA. (TIF)

Six genes (Basp1, Ncdn, Nrp-1, Brachyury T, CD155 and CD221) were selected for further confirmation of their differential expression between IVD regions (AF, NP) during aging (1 m, 12 m and 21 m) by RT-PCR. Differences in mRNA levels between AF and NP tissue were consistent with the results obtained through microarray analysis for all of the genes except Nrp-1 (Fig. 2). In immature (1 m) IVD, RT-PCR revealed that NP cells expressed higher mRNA levels of Basp1 (8.7-fold), Ncdn (10.1-fold), Brachyury T (4-fold), CD155 (6-fold), CD221 (5.2-fold), but not Nrp-1 as compared to AF cells. During IVD maturation (12 m) and aging (21 m), NP cells maintained this trend of higher expression for the aforementioned targets as compared to AF (Fig. 2), though the mRNA level of CD221 in aged NP (21 m) was similar to that of aged AF (Fig. 2 F). In addition, RT-PCR revealed that mRNA levels of Basp1, Ncdn and CD155 in aged NP (21 m) were ∼2–7-fold higher than that in mature (12 m) and immature NP (1 m) (Fig. 2A, B and E). However, mRNA levels of Nrp-1 and CD221 in aged NP (21 m) were roughly 4 to 6-fold lower than that in mature and immature NP (Fig. 2 C, F). Interestingly, the mRNA expression of Brachyury T in aged NP (21 m) was at the same level as in immature NP (1 m) although about 2-fold increase was found in mature NP (12 m) as compared to immature NP (1 m) (Fig. 2 D).

### Protein Expression of NP Markers during Aging

**Figure 3:**
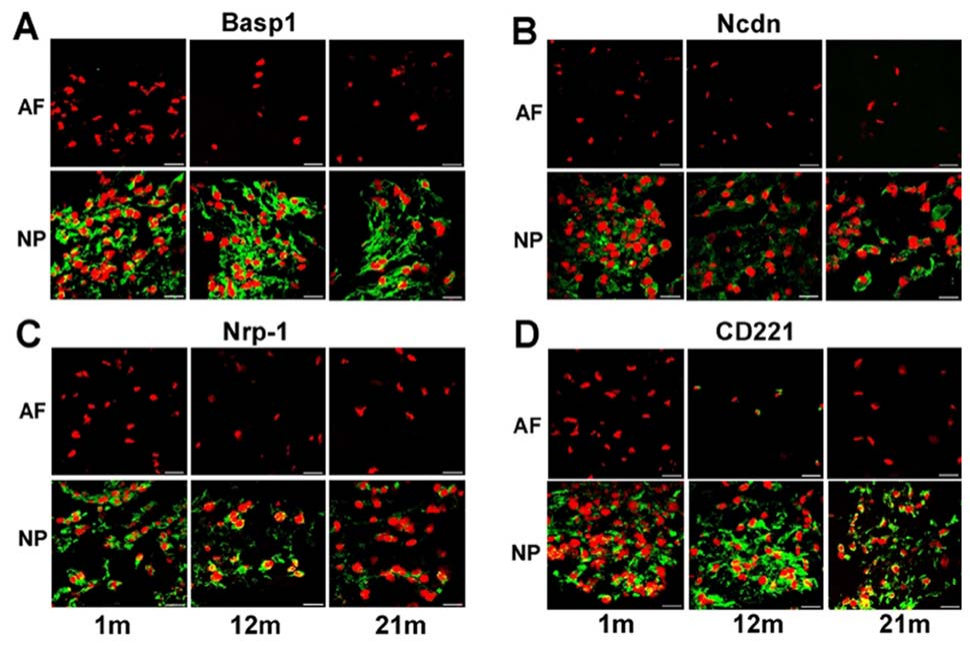
Immunostaining illustrated region-dependent and age-related changes in the expression of NP markers. (A) Basp1; (B) Ncdn; (C) Nrp-1; (D) CD221 in rat IVD tissues at different ages (1 m, 12 m and 21 m). Bar: 20 µm; m: month. (TIF)

Similar to gene expression, neuronal-related proteins (Basp1, Ncdn and Nrp-1), transcriptional factor (Brachyury T) and CD proteins (CD24 and CD221) were found to exhibit a strong tissue-specific expression in NP region as compared to AF cross all ages. Intense positive staining of Basp1, Ncdn and Nrp-1 was detected in cells from all NP tissues as a dense network-like appearance connected to cells in NP of immature, mature and aged tissues, whereas no positive staining was observed in AF tissue at any age (Fig. 3 A, B, C). Noteworthy, the differential expression of Nrp-1 between NP and AF detected only by microarray (Table 1) but not by RT-PCR (Fig. 2 C) was confirmed at the protein level via immunostaining (Fig. 3 C). A less intense staining for Nrp-1 was observed in aged NP (21 m) versus that in immature (1 m) and mature NP (12 m) (Fig. 3 C).

**Figure 4:**
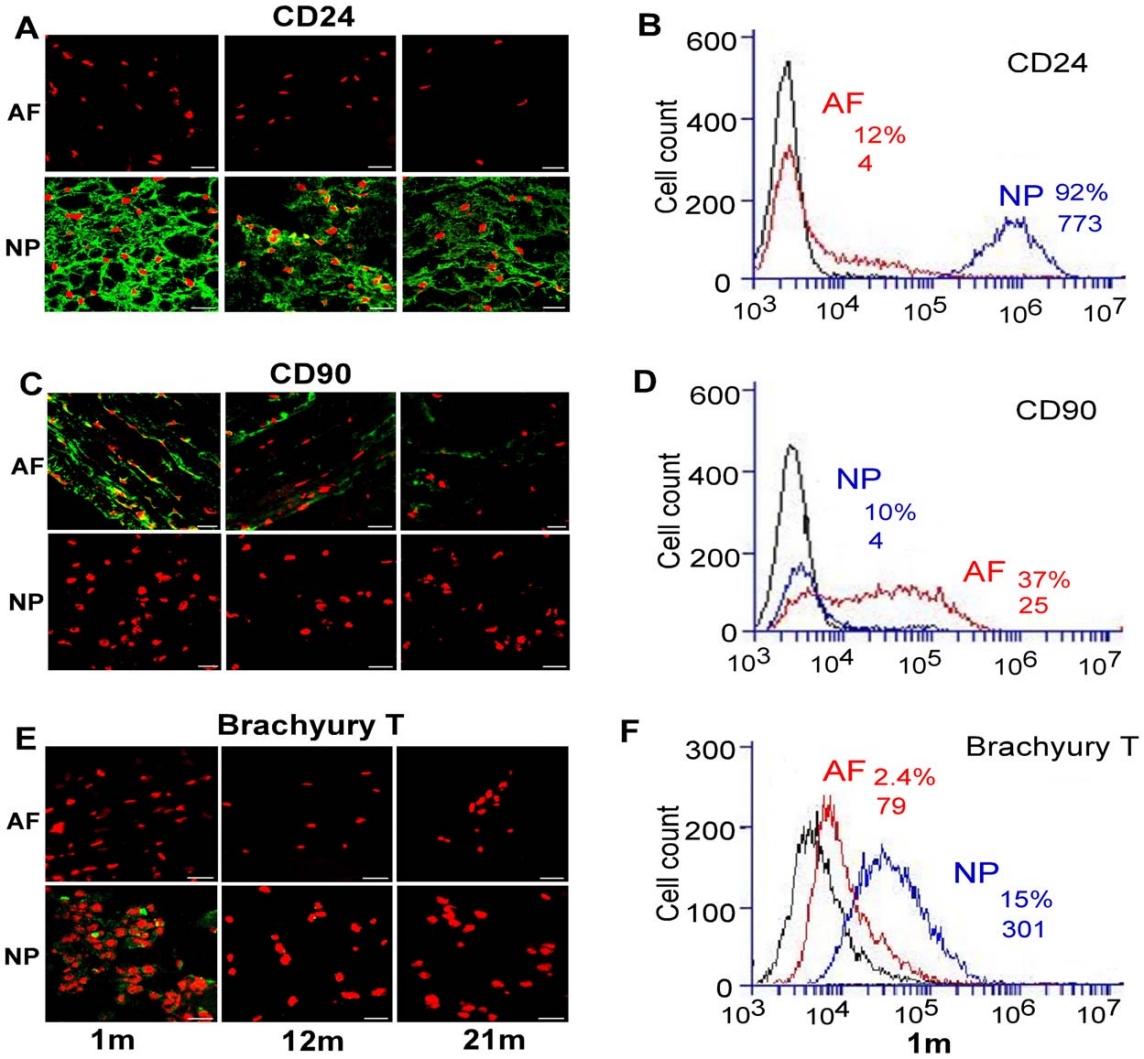
Immunostaining and flow cytometry detection for NP markers. Immunostaining (A, C, E) and flow cytometry (B, D, F) illustrated region-dependent and age-related changes in the expression of NP markers (A, B) CD24; (C, D) CD90; (E, F) Brachyury T in rat IVD tissues at different ages (1 m, 12 m and 21 m). Bar: 20 µm; m: month. Representative histograms of flow cytometry at left illustrate the relative fluorescence intensity of NP markers on X-axis for freshly isolated cells of 1 m rats (cell surface: CD24 and CD90; nucleus: T). The numbers appeared in each histogram indicate the percentage of positive fluorescence labeled cells and mean fluorescence intensity (MFI) for each cell type. (First left black line: isotype control, red line: AF cells, blue line: NP cells). (TIF)

For protein expression of cell surface receptors, a similar intense positive staining of CD221and CD24 protein was observed in NP tissues of all ages (Fig. 3 D and Fig. 4 A), and these protein expression patterns remained consistent with their NP tissue-specific gene expression (Table 1 and Fig. 2 F). In mature AF, CD221 was stained only slightly positive in some AF cells (Fig. 3 D). Conversely, a distinct pericellular staining of CD90 was observed in AF tissue with a declining trend during aging, while no staining was detected in NP of any ages (Fig. 4 C). Flow cytometry further confirmed that a higher percentage of immature (1 m) NP cells was labeled with high fluorescent intensity (92%, MFI: 773) for CD24 as compared to their AF counterparts (12%, MFI: 4) (Fig. 4 B). However, CD90 expressed higher in AF than in NP (AF: 37%, MFI: 25; vs. NP: 9%, MFI: 4) (Fig. 4 D).

Immunostaining revealed that transcriptional factor Brachyury T positively expressed only in immature (1 m) NP (Fig. 4 E). Flow cytometry further demonstrated that Brachyury T expression exhibited regional specificity, as higher levels were observed in immature NP cells (15%, MFI: 301) compared to that in the AF (2.4%, MFI: 79) (Fig. 4 F). These results in immature NP are thereby consistent with Brachyury T’s gene expression pattern (Table 1; Fig. 2 D). However, unlike gene expression, the protein expression was not detected in either mature or aged NP (Fig. 4. E).

## Discussion

The IVD undergoes tremendous cellular and functional changes with aging, including decreased cell number, and altered cell phenotype and matrix composition, which are generally implicated in disc degeneration [39], [40], [41]. However, the correlation between molecular phenotype changes of disc cells and the onset of disc degeneration has yet to be elucidated. Our current study discovered and confirmed a new set of NP-markers (Basp1, Ncdn, Nrp-1, CD24, CD155, CD221 and Brachyury T) and one non-NP marker (CD90) through a combination of tools including cDNA microarray, realtime RT-PCR, immuno-histochemical staining and flow cytometry analysis. Our findings also revealed significant age-related changes for many of these markers at both the mRNA and protein level during rat aging. These proteins not only can be used to define a distinct molecular phenotype for rat NP cells, but may also represent potential markers that define immature NP cell phenotypes in the human IVD.

A novel finding is that the neuronal-associated proteins, Basp-1, Ncdn and Nrp-1 more highly express in immature rat NP, suggesting their relationship to the notochordal-like characteristics of immature NP cells. Brain abundant membrane attached signal protein1 (Basp1) is a novel myristoylated calmodulin-binding protein found predominantly in neurons and the spinal cord, which participate in neurite outgrowth and synaptic plasticity [42], but may also be detected in several other tissues including spleen, kidney, and testis indicating its diverse functions [43]. A recent study also reported that Basp1 is a transcriptional cofactor of WT1 (Wilms’ tumour 1) to regulate organogenesis and lineage potential of blood cell [44], as well as a tumor suppressor for myc-induced oncogenesis [45]. In this study, we note Basp1 expression specifically in the NP region of IVDs across all age groups (1, 12 and 21 m) in rat. Interestingly, Basp1 increased at both mRNA and protein levels in the aged rat (21 m) as compared to immature and mature rat (1 m & 12 m). Minogue et al., also reported a slight increased mRNA expression of Basp1 in NP cells of bovine as compared to AF cells, while a significantly increased mRNA expression of Basp1 was found in degenerated human AF cells as compared to normal AF cells [33]. Neurochondrin (Ncdn), also named norbin in the rat, was first identified in mouse brain [46]. It is a cytoplasmic leucine-rich protein involved in neurite outgrowth and chondrocyte differentiation [47], [48]. Similar to Basp1, Ncdn strongly expressed in rat NP of all age groups (1, 12 and 21 m) at the gene and protein levels, yet its protein expression was not detected in rat AF of any age. Interestingly, mRNA levels of Ncdn in aged rats (21 m) were significantly higher than that in immature and mature rat (1 m & 12 m). The NP-specific and age related expression patterns of Basp1 and Ncdn indicate their possible functional relationships in NP development. Notochordal cells comprise the dominant cell population in the immature rat NP [12], [14] and play a role in spinal cord and vertebra development, in addition to patterning and differentiation of the IVD [49]. The data presented here suggests that Basp1 and Ncdn may be notochordal markers and also involved in NP maturation and age-related degeneration. Future studies will be necessary to explore their functional roles in IVD development and pathological disorders via knockout animal models of Basp1 or Ncdn.

Neuropillin-1 (Nrp-1) is a neuronal cell transmembrane glycoprotein that mediates neuronal guidance and angiogenesis [50], but can also be detected in many non-neuronal tissues [51]. In our study, decline of Nrp-1 was observed in aged rats (21 m), but not in mature and immature rats (1&12 m). This age-dependent expression of Nrp-1 may be associated with changes to its receptors (i.e. VEGF165R and emaphorins 3a) in aged rats. Prior studies revealed that Nrp-1 is a novel vascular endothelial growth factor receptor (VEGF165R) and modulates VEGF binding to VEGFR-2 to regulate VEGF-induced angiogenesis [52]. Nrp-1 also was found to bind with semaphorins 3a and to induce sensory axons to repel collapse of their growth cones [53]. Indeed, a recent study reported that a significant decrease of semaphorins 3a and Nrp-1 in the degenerate human IVD causes increased neural ingrowth [54]. Altogether, these results suggest that Nrp-1 may possess a regulatory role in disc degeneration. The results of real time RT-PCR for Nrp-1 could not confirm our finding for Nrp-1 by cDNA microarray, but the results of immunostaining for Nrp-1 protein expression were consistent with the results of the cDNA microarray. This may highlight an importance to confirm cDNA microarray data for accuracy through multiple assays at different expression levels (such as both mRNA and protein).

Brachyury T, a transcription factor essential for the genesis, differentiation and survival of mesoderm and notochord [55], [56], is known to be a specific marker for the notochord and notochord-derived tumors [57]. The notochord represents a crucial structure during embryonic development. The majority of notochordal cells die and are replaced by bone in the vertebral bodies and eventually formed NP cells in the intervertebral discs during embryogenesis [49]. However, still some notochordal-like cells could be detected in the immature nucleus pulposus of several species of animals [14], including humans [58]. Most of these cells gradually lose their notochordal cell morphology during aging [58]. In the rat, notochordal cells are the dominant cell population in immature tissue, while virtually no notochordal cells are present by 1 to 2 years of age [12], [39]. Our study with Brachyury T documented for the first time its gene and protein expression in NP of rat intervertebral disc during aging. Brachyury T mRNA expressed at significantly higher levels in NP than in AF for all age groups (1 m, 12 m, 21 m). However, its protein expression was only detected in NP tissue of 1-month old, but not 12- or 21-month old rat by immunohistochemical staining, and flow cytometry further revealed that only 15% of 1-month old NP cells expressed brachyury. This data supports that brachyury may be involved in notochordal cell differentiation and early stages of NP development. The age dependent expression of brachyury T is highly correlated with the disappearance of notochordal cell morphology in NP with aging. Future studies for the regulation of brachyury in NP development may reveal a molecular mechanism governing disc degeneration. Furthermore, we are currently investigating other NP-abundant transcriptional factors (NR3C2, SREBF2, KLF6, SREBF1 and RELa) identified by cDNA microarray for their functional control of notochordal cell changes during NP development.

Moreover, we are interested in cell surface receptors that may be NP cell markers. CD24, a glycosylphosphatidylinostitol-anchored cell surface protein, is expressed in neurons, preB cells, T cells, and several cancer cells [59] and functions in differentiation and activation of granulocytes and B lymphocytes [60]. A recent study showed that CD24 is expressed by rat NP cells of rat and human chordoma (notochodal tumor) [30]. We further confirmed that CD24 was strongly expressed in the immature NP (1 m) in a tissue specific manner through flow cytometry and immunohistochemical staining. Furthermore, CD24 continued to express in both mature and aged NP (12 m and 21 m). These findings indicate that CD24 may play a role in NP development and homeostasis. In contrast, CD90 (Thy-1), a cell-surface-anchored glycoprotein [61], has been found in many kinds of stem/progenitor cells [62]. CD90 was also reported in AF and NP cells of degenerated human disc [63]. Our results here note that the expression pattern of CD90 was AF-specific. Therefore, we propose that CD90 can serve as a non-NP phenotype of disc cell marker.

CD155 (poliovirus receptor, PVR), originally identified as the poliovirus receptor, is an Ig-like cell surface protein expressed on many cell types that has recently been discovered to have immune regulatory properties [64]. Most of the immunologic effects of CD155 are mediated by its interaction with DNAX accessory molecule-1 (DNAM-1) (also called CD226) or CD8/CD96 on the surface of leukocytes [65], [66]. The extracellular region of CD155 has been reported to bind to the extracellular matrix molecule vitronectin [67]. In this study, both cDNA microarray and real time PCR results displayed 4.86-fold and 6-fold increases respectively in mRNA levels in immature NP as compared to AF, and the mRNA levels were also increased in aged NP only. Because rat-specific CD155 antibody is unavailable, the protein expression of CD155 and its relationship to aging is still unknown.

CD221 (IGF-1R, insulin-like growth factor receptor-1), is a high affinity receptor for IGF-1. The functional receptor is a homodimer that comprise two subunits, α and β, which contain a kinase domain responsible for initiating a signaling cascade [68]. It has been reported that IGF-1R expressed more in young NP cells than in mature cells in bovine and rat [69], [70]. In addition, IGF-1R was also detected in degenerated human NP and inner AF [71]. Various studies showed that IGF is capable of enhancing proteoglycan synthesis in IVD cells [69], [70], [72] and exerts anti-apoptotic effects on human IVD disc cells by combining with its receptor, IGF1R [73]. Our study demonstrated that CD221 was highly expressed in immature NP (1 m) and mature NP (12 m), but decreased significantly in aged NP (21 m). These findings indicate that CD221 may be a phenotype marker for NP in the non-degenerate stage, and likely play a vital role in NP development through a special signaling cascade.

Finally, it is worthy to note that cDNA microarray with Affymetrix GeneChip technology has also been used for the gene expression profile in intervertebral disc cells of rat, canine and bovine in several previous studies [31], [32], [33]. However, we found only a few similar gene targets (such as CDH2, BASP1) listed in Table 1 were also reported differential expression between NP and AF tissue regions in these previous studies. It is possible that we have focused on finding new targets of cell surface receptors and transcriptional factors by using immature animals. Importantly, the RNA sample we used in experiment, was directly isolated from two distinct tissue regions (AF, NP) of IVD at different ages (immature and mature groups) immediately after sacrifice the animal, while RNA samples used in some of previous studies were from the cells of mature disc tissue by enzymatic digestion for at least several hours [31], [33]. Our method minimized the possible effect of experimental condition on gene expression changes for our interested targets and also may preserve all gene expressions at their levels of tissue and age in situ during the process of RNA isolation.

In summary, this study identified a set of novel markers including neuronal-related proteins, transcriptional factors, and a series of CD proteins for rat NP cells. Among these markers, the protein expression of Brachyury T is exclusively found in immature NP, but not in AF. Brachyury protein is also not detected in mature and aged NP or AF. This suggests that Brachyury (T) may serve as a specific marker for notochordal NP cells. Nrp-1 and CD221 proteins are expressed in NP with the trend of decline in aged NP but almost not found in AF. These age related expression patterns may also correlate to the degeneration of NP cells, suggests that Nrp-1 and CD221 may serve as markers for notochordal and mature NP cells. In addition, the protein expressions of CD24, Basp1 and Ncdn are exclusively found in NP of all ages and not in AF of any age, and their expression levels do not correlate with age-related degeneration of NP cells. Therefore, CD24, Basp1 and Ncdn may serve as general NP markers. In contrast, CD90 is only expressed in AF of all age with higher levels in younger age (1 m) and not found in NP of any age, suggests that it may serve as a marker for immature AF cells. Together, these age-related phenotype changes offer new insight for a molecular profile that may be used to characterize the NP. Such findings allude to a noteworthy correlation of cell-specific markers with disc aging and degeneration. Nevertheless, the functions of these markers implicated in NP development and differentiation still require further investigation.

## Materials and Methods

### Ethics Statement

All the procedures specified below were carried out in strict accordance with a protocol approved by the Duke University Institutional Animal Care and Use Committee (protocol number A202-09-07).

### IVD Tissue Harvesting

IVDs were harvested from the coccygeal spines of immature (Fisher 344, 1-month old, 1 m, n = 20), mature (12-month old, 12 m, n = 20) and aged (21-month old, 21 m, n = 20) rats within one hour of sacrifice (Duke University Vivarium). The disc were dissected and separated into zones of AF and NP according to their highly heterogeneous anatomical regions and distinct morphological appearance, where the AF is highly hydrated with concentric lamellar structure, and the gelatinous NP is clearly demarcated from the surrounding fibrous tissue as described previously [74]. AF and NP tissues were procured separately and processed for RNA isolation, cell isolation and immunostaining or histological evaluation as described below.

### RNA Isolation

Harvested AF and NP tissues were immediately flash-frozen in liquid nitrogen, then pulverized and homogenized in TRIzol reagent. Total RNA was extracted using the RNeasy mini kit plus DNase I digestion (Qiagen, Valencia, CA) [27]. For the cDNA microarray study, tissue was pooled from all discs across four animals to collect sufficient RNA for one sample and a total of four RNA samples (n = 4, 16 animals) for each tissue type (AF, NP) and age (1 m and 12 m) were analyzed. For realtime RT-PCR, another set of RNA samples was generated without pooling across animals and a total of another 4 RNA samples (n = 4, 4 animals) for each tissue type (AF, NP) of different ages (1 m, 12 m and 21 m) were analyzed. All RNA sample were strictly evaluated to ensure their integrity (the ratio of 28S:18S RNA = 2∶1) and purity (OD260/OD280 = 1.8–2.0) using an Aligent BioAnalyzer (Agilent Technologies, Clara, CA) according to the manufacturer’s instruction.

### cDNA Microarray

A total of 16 RNA samples (n = 4 each for AF and NP tissues of 1 m and 12 m rats) were hybridized to a rat GeneChip® (rat genome 230 2.0 Array, Affymetrix, Santa Clara, CA). 5 µg of total RNA was used for synthesis of cDNA via reverse-transcription. The cDNA was biotin-labeled, fragmented and hybridized to the GeneChip®. Afterwards, the arrays were washed, stained with streptavidin phycoerythrin and imaged for analysis (Affymetrix). All raw data was normalized and scaled using recommended microarray analysis protocols (Partek® Genomics Suite, Partek Incorporated, St. Louis, MO). Significant differences in expression between two different regions (1 mNP vs 1 mAF) and different ages (1 mNP vs 12mNP) were evaluated via one-factor ANOVA (unequal variances, Pearson (Linear) Correlation, Regression one-way). The identified targets were chosen based on a significance level of 0.01 and fold-differences greater than or equal to two fold between groups. This manuscript reports select targets related to cell surface receptors, transcriptional factors and neuronal-related proteins.

### Real-time RT-PCR

To validate the findings of novel targets from the cDNA microarray, real-time RT-PCR was performed on the iCycler iQ system (BioRad, Hercules, CA). Each target gene consisted of two rat-specific PCR primers and one fluorescently labeled intron-spinning probe from Applied Biosystems (Foster City, CA, Table 2). Real-time RT-PCR conditions were used as described previously [75], and the housekeeping gene β2-microglobulin served as an internal control. Duplicate PCR reactions were performed for each RNA sample and the internal control. Differences in ΔCt (Ct of target – Ct of β2-microglobulin) values between NP and AF or among age groups were analyzed for significance using a two-factor ANOVA (StatView, SAS Institute, Cary, NC). Fold-differences of relative mRNA level (2**^−^** ^ΔΔCt^) between NP and AF or among age groups were reported if greater than or equal to two fold (p<0.05).

**Table 2:** Realtime PCR probes and primers of NP-markers (from Applied Biosystems) and corresponding antibodies for protein analysis.

Target	Probe/primers	Antibody		
	Order number	Order number	Host/type	Isotype control
		(vender)	(application)	(vender)
BASP1	Rn 03035021	AB9306	Rb/Polyclonal	None
		(Millipore)	(IHC)	
Ncdn	Rn 00585542	AB88877	Ms/Polyclonal	Ms IgG
		(Abcam)	(IHC)	(BD
				pharmingen)
Nrp-1	Rn00686102	2621–1	Rb/monoclonal	Rb IgG
		(Epitomics)	(IHC)	(Epitomics)
CD221	Rn00583837	SC-713	Rb/polyclonal	None
		(Santa Cruz)	(IHC)	
Pvr/CD155	Rn 00567148	NA	NA	NA
BrachyuryT	Rn 01527353	SC-166962	Ms/monoclonal	Ms IgM
		(Santa Cruz)	(IHC & FC)	(BD
				Pharmingen)
CD24	ND	551133	Ms/monoclonal	Ms IgM,*k*
		(BD	(IHC & FC)	(BD
		Pharmingen)		Pharmingen)
CD90	ND	MCA47RT	Ms/monoclonal	Ms IgG1
		(Serotec)	(IHC & FC)	(Millipore)

NA: not applicable, no commercially available rat-specific anti-CD155; ND: PCR not determined; Rb: rabbit; Ms: mouse; IHC: immunohistochemical staining; FC: flow cytometry.

### Hematoxylin and Eosin (H&E) and Safranin O Staining

AF and NP tissue samples from rat discs were embedded in Optimal Cutting Temperature (OCT) compound (Sakura Finetek, Torrance, CA), flash-frozen in liquid nitrogen, and stored at −80°C until cryosectioning. 7 µm-thick sections were fixed in 10% neutral buffered formalin (Azer Scientific, Morgantown, PA, USA) for 10 minutes, washed in 1% lithium carbonate solution (Mallinckrodt Chemicals, Phillipsburg, NJ), and stained with 0.5% safranin-O solution (Sigma, St. Louis, MO) for 60 seconds. Samples were rinsed with distilled water and counterstained with Mayer’s Hematoxylin (Sigma) to visualize individual cells. After serial steps of dehydration, sections were then mounted with histological mounting medium (Permount, Fair Lawn, NJ) and visualized with light microscopy for staining of extracellular matrix. A separate set of tissue sections were used for routine H&E staining (Sigma) to visualize the cell structure. Both total cell number and cells with vacuole-like structure were counted manually from eight images randomly selected in NP regions of rat discs at different ages, then used to determine the average percentage of cells with vacuole-like structure (n = 8 per rat, 3 rats per age group). Statistical analyses were used to detect any significant differences (p<0.01) in the percentage of cells among age groups using a one-factor ANOVA (StatView).

### Immunohistochemical Detection

Frozen tissue sections were fixed and then incubated with specific anti-rat antibodies for select NP markers (Table 2). To evaluate the expression of Basp1, Brachyury T, Ncdn and Nrp-1, tissue sections were fixed in acetone for 10 min at −20°C, permeabilized with 0.2% triton (Sigma) for 10 min at room temperature, and subsequently incubated with a blocking solution (3.75% BSA/5% goat serum, Zymed, Carlsbad, CA) for 30 min. Next, the sections were incubated for 2 hr with primary antibodies (Table 2). To detect CD proteins, sections were fixed in 4% formaldehyde (Electron Microscopy Sciences, Hatfield, PA) for 10 min at room temperature, blocked by the same blocking solution, and incubated with primary antibodies (Table 2). Control sections were incubated with only blocking solution or appropriate mouse or rabbit IgG isotype control antibodies (Table 2). All sections were incubated with appropriate secondary antibodies (AlexaFluro 488, Molecular Probes, Eugene, OR) for 30 min in blocking solution, counterstained with propidium iodide (0.2 mg/ml, Sigma) to label cell nuclei and imaged using confocal laser scanning microscopy (Zeiss LSM 510; 20x NA 0.5 and 63x water immersion NA 1.2 objectives; Carl Zeiss, Thronwood, NY).

### Primary Cell Isolation and Flow Cytometry

AF and NP cells were freshly isolated with a sequential pronase-collagenase digestion [75] from 1-month old rat tail IVD samples and suspended in cell culture media (Ham’s F-12 medium, Invitrogen Life Technologies, Carlsbad, CA) supplemented with 10% fetal bovine serum (FBS, HyClone, South Logan, Utah), 100 U/ml penicillin, 100 µg/ml streptomycin and 1 µg/ml fungizone (Gibco, Grand Island, NY). After a two-hour recovery period in the culture media at 37°C, cells (0.2–0.5×10^6^) were incubated for 0.5 hr with monoclonal antibodies against rat CD24, CD90 and Brachyury T using appropriate isotype controls described in Table 2. The cells were then labeled with AlexaFlour 488 (Invitrogen, Eugene Oregon) conjugated secondary antibody. For Brachyury T, the cells were permeablized with 0.1% saponin (EMD Chemicals, San Diego, CA) before immunostaining. The percentage of cells with positive proteins (%) and the mean fluorescence intensity (MFI) were quantified via flow cytometry (Accuri C6, BD Accuri Cytometers Inc., Ann Arbor, MI).
